# The Smart in Smart Cities: A Framework for Image Classification Using Deep Learning

**DOI:** 10.3390/s22124390

**Published:** 2022-06-10

**Authors:** Rabiah Al-qudah, Yaser Khamayseh, Monther Aldwairi, Sarfraz Khan

**Affiliations:** 1 Department of Computer Science and Software Engineering, Concordia University, Montreal, QC H3G 1M8, Canada; r_alquda@encs.concordia.ca; 2 College of Technological Innovation, Zayed University, Abu Dhabi 144534, United Arab Emirates; monther.aldwairi@zu.ac.ae; 3 Faculty of Computer and Information Technology, Jordan University of Science and Technology, Irbid 22110, Jordan; 4 ICT, Algonquin College, Ottawa, ON K2G 1V8, Canada; drkhansarfraz@gmail.com

**Keywords:** smart city, deep learning, zoning, transfer learning, images, automation

## Abstract

The need for a smart city is more pressing today due to the recent pandemic, lockouts, climate changes, population growth, and limitations on availability/access to natural resources. However, these challenges can be better faced with the utilization of new technologies. The zoning design of smart cities can mitigate these challenges. It identifies the main components of a new smart city and then proposes a general framework for designing a smart city that tackles these elements. Then, we propose a technology-driven model to support this framework. A mapping between the proposed general framework and the proposed technology model is then introduced. To highlight the importance and usefulness of the proposed framework, we designed and implemented a smart image handling system targeted at non-technical personnel. The high cost, security, and inconvenience issues may limit the cities’ abilities to adopt such solutions. Therefore, this work also proposes to design and implement a generalized image processing model using deep learning. The proposed model accepts images from users, then performs self-tuning operations to select the best deep network, and finally produces the required insights without any human intervention. This helps in automating the decision-making process without the need for a specialized data scientist.

## 1. Introduction

A smart city is a city that utilizes a set of the state-of-the-art technologies—information and communication technologies, to provide better services to its citizens and users. This general definition opens the door for many stakeholders to be involved in the design of smart cities, such as computer scientists, software engineers, business managers, urban developers, urban planners, and city officials. It is indeed hard to provide a unified framework for smart cities, and it is even harder to measure the success of a smart city in delivering its vision. The modern concept of smart cities evolved from the very first initiatives toward digital cities in the 1990s [1]. Furthermore, evolved through the years to a city that utilizes the emerging Internet of Things (IoT) technologies to achieve the smart city strategic objectives [2].

The existence of suitable technological infrastructure is vital to the success of any smart city project; hence, most projects were designed from a technological point of view and were implemented to deliver technological solutions to overcome some of the current limitations of traditional systems. However, considering the technological components only in the design of a smart city framework is inadequate. Here, we identify some issues that need to be handled in terms of both the people and the institution’s point of view:

1. Understanding the people’s needs and their backgrounds (such as cultural, political, economical, and social aspects).
2. Understanding the role of communities and their distinct features.
3. Limited transparency and accountability in the government.
4. Lack of qualified human resources.

Despite their importance, the aspects of people and institutions have not been fully considered in the ongoing smart cities projects. Hence, there is a need to build a more comprehensive framework for a smart city that has the following features:

1. Adaptability: the framework should be flexible to accommodate the differences among different cities.
2. Completeness: it must consider all aspects of a smart city, rather than being designed for a specific project/domain.
3. Scalability.
4. Sensitivity to people’s and institutions’ requirements and challenges.

At the present time, global cities are competing in building a “smart” image to attract new businesses, talents, and customers. This high competition has managed to attract young professionals who are seeking better opportunities and a modernized lifestyle. However, the success of one city in attracting newcomers is usually at the expense of other cities. Moreover, there is a worrying increase in the urbanized global population (currently estimated to reach 70% by 2050 [3]). Recently, the “smart city” theme has emerged as a lucrative trend in promoting cities to the general public as well as businesses.

Existing smart city solutions are focused on developing ICT-based solutions to improve existing systems [4]. Hence, this set of ambitious and independent solutions focuses on efficient operations rather than the overall modern urban planning policies. It fails to consider high-level global challenges such as affordable housing and income inequality. Therefore, most of the smart city work is merely an attempt to upgrade existing cities.

ICT-based solutions, with the emergence of data-hungry technologies such as the Internet of Things (IoT), cloud computing (CC), and wireless sensor networks (WSNs), are all about collecting, managing, processing, and delivering data using the existing and expanding networking infrastructures.

Although these solutions provide easy access to many services as well as more educated decision making, they fail to consider many other aspects such as the social and environmental aspects, as well as the interactions/relationships between these aspects. For example, modern ICT solutions rely heavily on the usage and spread of smartphones. Such trends are driving individuals of the same household away from each other, eliminating or at best minimizing social interactions. For example, it is common now for individuals to watch their shows separately using their smartphones, which increases power consumption and, hence, affects our global environment. Smart solutions are not deemed beneficial if they are not used smartly. These interactions are not captured by the current smart city solutions. A better understanding of the various elements of a smart city (and how they interact with each other) is essential to steer it in the right direction. A smart city must be aware of its citizens’ and businesses’ real needs and be able to accommodate these needs without altering the social-economical-environmental-cultural fabrics of the city. In particular, a smart city must now to face environmental challenges; it is not merely a lucrative business venture.

Despite the importance of solving existing urban challenges, this work recognizes that another important direction for a smart city is the design and realization of a new truly smart city from scratch. In fact, several projects have been designed in this direction, and more detailed examples are presented in Section 2.

To highlight the importance and usefulness of the proposed framework, we designed and implemented a smart image handling system targeted at non-technical personnel, namely, the image classification framework (ICF). We note that to facilitate the decision-making process in a smart city context, a considerable amount of data needs to be collected, processed, and analyzed. A key form of such data is images. Engineering imaging data to extract useful insights can be a hectic process, and it requires specialized data scientists to perform it. The high cost, as well as security and inconvenience issues, may limit cities’ abilities to adopt such solutions.

Therefore, this work also proposes to design and implement a generalized image processing model using deep learning. The proposed model accepts images from users, then performs self-tuning operations to select the best deep network, and finally produces the required insights without any human intervention. This helps in automating the decision-making process without the need for a specialized data scientist. Hyperparameter tuning such as batch size and input image resolution is a hectic process, and eliminating the human intervention can greatly reduce the cost and minimize errors. Moreover, the proposed system utilizes concepts such as transfer learning (TL) to increase the system’s accuracy. The system is also a flexible one that adapts based on the existing infrastructure to perform the given tasks (such as available hardware).

The main contributions of this work are:

1. A highlighting of the importance of understanding the transactions of existing solutions/cities to better design new smart cities.
2. A review of the literature on new smart cities.
3. A proposal of a new framework to design new smart cities.
4. A proposal of a new framework for the ICT infrastructure needed to support the new design.
5. The design and implementation of a smart image handling system targeted at non-technical personnel, namely, image classification framework (ICF).
6. In ICF, the framework considers five different deep networks and it was tested in three different datasets.

The rest of this paper is organized as follows: Section 2 presents and highlights the definitions of a smart city. Section 3 reviews some of the existing and related works. Section 5 presents the proposed smart city zoning framework. Section 6, Section 7 and Section 8 present the image classification framework (ICF), the dataset used, and the deep network models, and depicts the obtained results from ICF. Finally, the paper is concluded in Section 9.

## 2. Smart City

The concept of a smart city (also referred to as a sustainable smart city) is being widely used by both the public and the private sectors as a promising vision to enhance the quality of life and conduct of businesses.

The idea of a smart city emerged more than 15 years ago as a promising solution to overcome existing cities’ challenges and modernize our lifestyle [5]. Early visions of smart cities were promising and set to alleviate the burdens of urbanism, enhance the quality of life, and sustain the environment. Despite the many initiatives and projects toward smart cities, the current work falls short in delivering the promised “smart city”, due to:

1. Unrealistic visions of a smart city.
2. Unrealistic understanding of information and communication technology (ICT) capabilities and limitations. ICT is the prominent enabler of a smart city.
3. Some of the projects and initiatives were merely celebratory and for public relations purposes to promote someone or some image rather than realistic projects.
4. Most of the projects were corporate projects rather than public projects. This particular point is not meant to undermine the importance of the private sector, but rather to highlight the “universality” of the smart city concept. All stakeholders of the city (e.g., government, citizens, and businesses) must be involved in the design and implementation of these projects. The solely business-based solutions are more likely to lack the inclusion of other stakeholders’ visions in their problem formulation (i.e., identifying the essential problems facing cities) and in their solutions.

Most of the prior and current smart cities projects have been driven by technology-based companies, and they lack the perspective of other important stakeholders in their solutions [6]. The dynamic nature of cities, along with social, economical, cultural, and political differences, makes it impossible to have a standard framework for smart cities that is suitable for all cities.

Several standardization bodies have attempted to produce a set of standards for smart cities, such as the British Standards Institute (BSI), the International Standards Organisation (ISO), and the International Telecommunication Union (ITU) [7]. However, smart city standards are categorized into:

1. Strategics: these standards tend to describe the management side of a smart city, and do not focus on the technical aspects. They consist in a high-level description of the strategy, plans, and objectives of a smart city.
2. Process: these standards define and describe the data flow in a smart city.
3. Technical: these standards define the best practices to deploy and use technical infrastructure (such as communication networks and devices) to implement a smart city.

The most common components in any smart city framework are:

1. Technology: which technology is best to use, whether it is hardware or software;
2. People: individual needs and satisfaction are key components in defining a smart city and measuring its success;
3. Institutions: government offices, organizations, and policy makers. These institutions provide the required leadership and governance to smart cities.

As mentioned above, the term “smart city” is not well defined in the sense that no universally agreed-upon definition exists for it. Here, we summarize some of the common definitions of the term in the literature in Table 1.

## 3. Literature Review

City planning is a challenging and complex task. It involves many elements and requires careful planning and coordination between all stakeholders. It is usually carried out by the government and specialized entities. However, recent trends have emphasized the importance of the inclusion of all stakeholders in this process. Such involvement should result in a more feasible city plan; however, it may add to the complexity of this process. The work in [14] highlights this issue and proposes a new framework that considers both the smartness dimensions and the stakeholder and tries to map these two factors to minimize the complexity of the problem. The work in [15] discusses the potential involvement of the citizens in the design of the smart city rather than relying only on the technological aspects of the smart city design. The authors address the challenges of the so-called Citizen Design Science, such as high cost and low/poor representation of the citizens, by proposing to use an interactive online tool for citizens’ participation. There are two possible approaches to designing a smart city: a bottom-up approach and a top-down approach [16]. We note that the bottom-up approach is more suited to existing cities, where it is beneficial to involve the citizens in designing smart solutions to overcome existing challenges. For example, the city of Amsterdam in the Netherlands adopted this approach in its smart city initiative and focused on behavioral change and user involvement rather than technological advancements [5], although technology played a major role in the adopted initiatives in the city. The concept of living labs was used to test and evaluate the proposed solutions. The concept of living labs has also een adopted by many researchers. For example, the work in [17] proposed a living lab model to design smart city applications that satisfy two conditions: (1) citizens’ involvement in the design, and (2) environmentally aware applications. The top-down approach is more suited to the development of new cities. Such an approach has been adopted in designing the cities of Masdar in the UAE and Songdo in South Korea.

The rest of this section is divided into two parts. The first part reviews frameworks in the literature at the strategical level, and the second part reviews frameworks in the literature at the technical level.

### 3.1. Service-Level Frameworks

The work in [1] proposed a smart city framework that aligns the collected data from the smart city infrastructure (such as sensors) and the citizens’ requirements. This alignment is achieved through the introduction of a data model integration layer (DMIL). This layer facilitates information sharing among the different cities’ service providers. To accommodate citizens’ requirements, the authors introduce a new layer, namely, the smart cities integration layer (SCIL). The citizens’ requirements were derived from the Classification of the Function of Government (COFOG) set by the United Nations.

The authors in [18] proposed a high-level framework for a smart city based on a set of enablers extracted from the literature. The proposed framework was designed for developing countries (in particular, India) by identifying key enablers along with the country’s key economical indicators and recent smart city projects. The authors depended on a panel of experts to evaluate and weigh all obtained enablers. The study revealed that innovative construction techniques, supportive government policies, and advanced information and communication technology are the key enablers for constructing a smart city in developing countries. A similar approach was taken in [19], in which the authors used the crowd-sourcing technique to collect 19 ideas to build a smart city transformation framework.

### 3.2. Technical-Level Frameworks

A data-driven framework is proposed in [20] over a distributed system to facilitate data sharing among the city services. The proposed solution utilizes IoT and cloud technologies to support its applications. It consists of two main components: large-scale data stream processing modules and adaptive decision support modules. To increase the feasibility of this framework, the authors developed a context-aware real-time travel planner application.

The authors in [21] proposed a smart city framework to handle the large amount of data generated from all sources and to achieve quality of experience (QoE) for the applications/services. For this, a three-plane data-based framework is proposed, consisting of a storage plane, a processing plane, and an application plane. Moreover, a deep learning network is devised to extract accurate data for the end users. A high QoE results were obtained using a simulation for accuracy, precision, and recall metrics.

Santana et al. [22] identify four main enablers for a smart city: big data, cloud, IoT, and cyber-physical systems. They reviewed key smart city frameworks in the literature and identified the main functional and non-functional challenges of a smart city. Then, they proposed a reference software platform to implement effective smart city applications.

## 4. Smart City Elements

At the present time, the word “smart” can be combined with any existing system in the city. In a smart city, all services provided by the city must be smart. Examples of these services include, but are not limited to, predictive policing, transportation, health, education, waste management, water supply, utilities supply and management, and law enforcement. We note that there has been a substantial amount of work and projects to enhance the individual services provided to the citizens to reduce cost, save energy, and save time. However, a truly smart city must provide a unified framework that manages and controls these services in a smart fashion and facilitates the integration, if possible, between these services. It became evident that one service may have a substantial influence on the quality of other services. For example, transportation services significantly influence education and health services.

Allam and Newman [23] proposed a rather high-level framework for a smart city that is based on cultural, metabolic, and governance dimensions rather than an ICT-based framework that favors developing new smart cities rather than bringing smartness to old/existing cities.

Now, it is important to tackle the main elements of any city and discuss how each of these elements is related to the proposed design. First, we need to highlight the main trends in future smart cities and then discuss how they may impact each element individually: Here, we identify the following main trends:

1. Less mobility: in the future, it will be easier for citizens to perform their jobs’ related tasks from home. Furthermore, online learning is expected to be adopted by more educational institutions at all levels (e.g., schools and universities).
2. An innovative and efficient home delivery system: with the lack of mobility and the recent trends of online shopping and at-home lockdowns, more energy-efficient and fast delivery systems will be needed in the future.
3. More spread of renewable energy sources: more and more renewable energy initiatives are being adopted by both the public and the private sectors. This will contribute significantly to the green vision.
4. More ICT-based solutions: technology is advancing at an unprecedented pace, and the rise of deep learning at the software level, as well as the rise of IoT devices and virtual and augmented reality devices at the hardware level, are projected to provide seamless solutions to citizens and open the door to new innovative green solutions to existing problems.

Now, we identify the main future trends in some key elements of smart cities that will shape the future vision of smart cities.

**Transportation**. This plays a vital role in any community (large or small). It is the means by which residents can reach their destinations and of transporting goods and products from one place to another. Hence, transportation challenges are vast and have a direct and indirect influence over all other elements. One example is the adaptation of electric and environment-friendly vehicles to reduce their environmental impact and reduce the consumption of fossil fuels. Moreover, distant learning and a flexible work environment will result in a reduced usage of transportation systems. Hence, IT can play a role in smart traffic management systems by managing the current transportation systems to reduce congestion and transportation times [24].

**Waste Management.** It is essential for modern smart cities to reduce their amounts of waste by relying more and more on organic and locally grown products. Moreover, recycling will play a major role in reshaping waste management systems. However, there still will be a need to manage non-organic wastes. Modern waste management systems must support the following features: ease of access and use by citizens, energy efficiency, and support for recycling in an automated fashion [25].

**Education.** Online and blended education are gaining more popularity for many reasons, such as health concerns, traffic congestion, schedule flexibility, and economical matters. This shift has gained tremendous momentum recently due to the recent outbreak of the Coronavirus (COVID-19), and it may have a direct and indirect impact on many areas such as bullying, transportation, and public health [26].

**Energy.** There are two key directions in this domain:

1. A greater reliance on sustainable energy from renewable resources such as solar and wind [27,28].
2. A reduction in energy consumption through a better management of available resources, and this direction is more apparent with the rise of smart energy grids and their potential positive impact in reducing the amount of consumed energy [29].

## 5. Smart City Framework

It is acceptable to have a unified and standard infrastructure for ease of planning and execution; however, the city must provide room for a sense of individualism and uniqueness. Even “smart” cities must have their own identity. The idea of designing a new city is well justified to accommodate population growth and to open the door to more economical opportunities. A well-designed new city is expected to have economic and environmental impacts. A city consists of constrained physical parameters inhabited by its citizens and governed by its political structure. Most of the work in smart cities has targeted the optimal utilization of the city’s resources while taking into consideration the physical constraints. Most of these have been mere pilot projects to tackle certain domains rather than a holistic approach. We need a deep understanding of the activities performed in the cities to come up with novel designs for new smart cities. Therefore, in this work, we focus our attention on the design of a new city rather than on finding solutions to address the shortcomings of the existing cities. A goal-oriented design is proposed that considers the following key principles:

1. The city must have a clear and well-defined vision and objectives.
  (a) New country’s capital.
  (b) Ease the burden from big cities.
  (c) New attraction/innovation sites.
2. The defined objectives must be measurable.
3. The city must have a suitable and realistic economical plan that defines:
  (a) The city’s economic functions;
  (b) Sustainable financing and resources;
  (c) Affordability.
4. Cultural identity.
5. A futuristic digital master plan to avoid existing issues inherited from existing cities, and to avoid the costly solutions associated with these issues.

**Figure 1:**
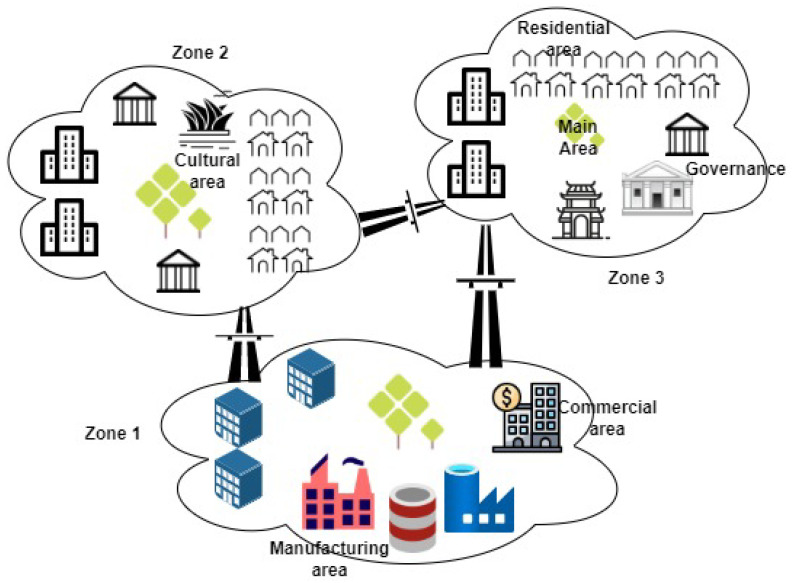
Zoning framework for a new smart city.

To achieve this, we derive the following rules: shorten the supply chain, and focus on reducing, reusing, and recycling principles to tackle issues such as waste management; use zoning design in which the city is divided into sustainable and somewhat self-sufficient zones; the design must eliminate or minimize the need for a central zone (e.g., a city center) to avoid congestion and centrality problems. The zoning framework is depicted in Figure 1. The zones share some common attributes, such as:

1. Government body;
2. Residential housing;
3. Commercial facilities;
4. Transportation facilities;
5. Green area;
6. Waste management system.

Additionally, each zone might have its own identity and attributes, in particular, a cultural identity; for example, it may have specialized restaurants/cuisines.

**Figure 2:**
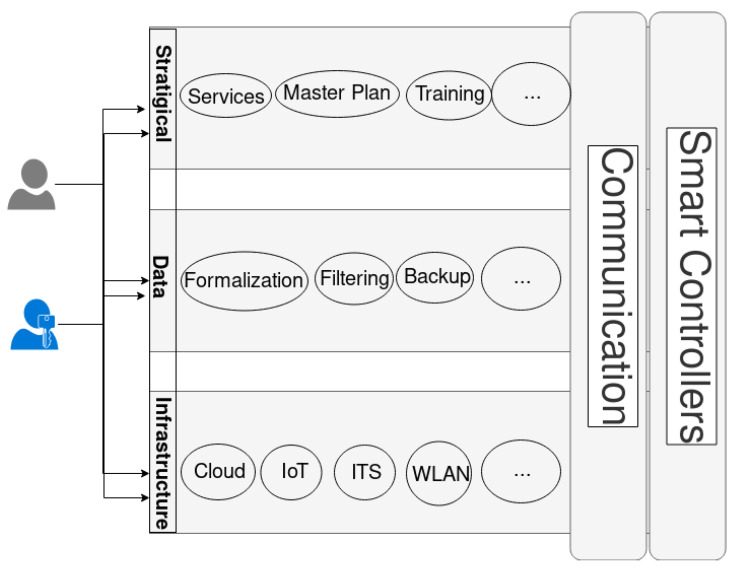
General framework of a smart city.

To best accommodate all these requirements, we propose a hybrid modular and layered framework design as shown if Figure 2. The framework consists of three planes, as follows:

1. Strategic plane;
2. Infrastructure (technological) plane;
3. Data plane.

Each plane will consist of as many modules as needed to support its operations. Each plane must support at least one communication module that interacts with the other two planes using message passing.

In this framework, and in addition to the three proposed planes, smart controller modules are integrated into each plane to account for the intelligent components in the system. We define the following smart controllers for each plane as:

1. Strategic controllers:
  (a) Rules and objectives extractor;
  (b) Objectives evaluators.
2. Data controllers:
  (a) Data fusion;
  (b) Data analysis.
3. Infrastructure controllers:
  (a) Network selector.

## 6. Image Classification Framework (ICF)

**Figure 3:**
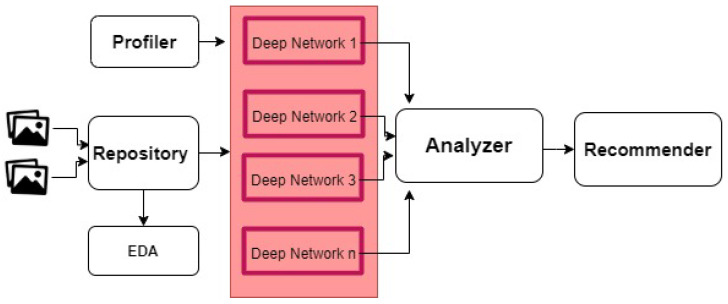
Main architecture of the smart-deep system.

A key challenge for a successful adaptation of the smart city vision comes from the readiness and acceptance of this vision by non-technical officials and employees of the city. For employees to accept such a change, they must become an integral part of this vision. One way to address this challenge is by designing a system with a high utility and usability that produces in-depth analyses and generates insights from massive amounts of collected data. In addition to the benefits of the active engagements of all stakeholders, this design will achieve a cost reduction in the overall system as the need to hire specialized data analysts diminishes. Deep learning is gaining popularity in image classification tasks, as it can achieve high-performance results by correctly identifying objects of various shapes and conditions even in complex environments. In practice, many deep learning architectures exist, and they usually vary in performance depending on the complexity of the classification task and domain. Specialized deep network engineers are typically employed to handle this task and utilize the right tools to produce good classification results. For this, we propose an automated image classification system using deep learning in which the system accepts raw images from users, performs the classification task using several well-known deep learning algorithms, and automatically measures their performance in terms of many metrics (such as accuracy, F1-score, and recall). Figure 3 depicts the general architecture of the proposed framework. In this architecture, the user first feeds the available images to the repository component of the framework. Next, the images are emitted to a set of deep networks sequentially. Each deep network trains on the given training set before it provides its results to the analyzer component. In this framework, the following deep neural networks were utilized, due to their good performance in classification:

1. Xception [30].
2. DenseNet121: DenseNet121 is utilized for the low memory profile, and DenseNet169 for the high memory profile [31].
3. MobileNet: MobileNetv2 is utilized for the low memory profile, and MobileNet is utilized for the high memory profile [32].
4. ResNet: ResNet50 is utilized for the low memory profile, and ResNet101 is utilized for the high memory profile [33].
5. VGG: VGG16 is utilized for the low memory profile, and VGG19 for the high memory profile [34].

The analyzer compares and analyzes all model-level results from all networks and submits its findings to the recommender component. Finally, the recommender selects the “best” results for the user. The profiler component performs the memory profiling task based on the user’s choice.

The proposed framework offers many functionalities, as follows:

1. Exploratory data analysis (EDA). This feature allows non-technical users to better understand the used dataset, as it provides statistical and visual insights about the instances of the training, validation, and test sets. The EDA module produces three sets of insights:
  (a) Statistical insights of the training, validation, and test sets in terms of the total number of instances, and a number of classes in each of the dataset portions. At this point, the framework verifies that the number of classes in all dataset portions match, in order to avoid fatal errors during the training phase.
  (b) Visualization of the training and test sets are depicted in histogram figures to provide insights into the distribution of instances and the level of balance among the dataset classes. Moreover, random samples of the dataset instances are displayed in order to help explore and verify the nature of the data and discover possible patterns among the instances.
  (c) The Shannon entropy score (SES) is also calculated twice at this step, for the training set and the test set, to measure the level of balance in each set. Typically, SES is used to measure randomness in a given dataset. In this context, we redefine and utilize SES to measure how a given dataset is balanced. Data balance is a key factor in the success of any neural/deep network in predicting/classifying tasks. An unbalanced dataset tends to increase the number of false predictions and classifications. The SES value ranges from zero to one, and a value closer to one indicates a more balanced dataset. The SES is calculated using Formula (1) for a dataset of *n* instances that consists of *k* classes of size $c_{i}$:
    (1) $$H=\frac{-\sum_{i=1}^{k}\frac{c_{i}}{n}*log\frac{c_{i}}{n}}{logk}$$
2. Transfer learning (TL). The main goal of TL is to benefit from other datasets to enhance the target neural network performance. In fact, the framework supports two options in terms of utilizing TL:
  (a) Option 1: The user can either choose to initialize all neural network weights randomly and train them from scratch by choosing the “No TL” option, or utilize TL by initializing the weights to ImageNet pretrained weights.
  (b) Option 2: If the user opts to utilize TL, then two more options are available: fine-tuning the pretrained weights or freezing them. The user is encouraged to choose the freezing option in case of low memory availability due to its lower consumption.
3. Determinism. Deep neural networks use randomness by design. This randomness causes a lack of reproducibility. To address this issue, the proposed framework gives the user the option to utilize deterministic neural networks that utilize the same random numbers [35].
4. Memory profiles. Since the proposed framework deals with images, which usually require more memory, the user can choose, based on hardware availability and problem complexity, one of the following two memory profiles:
  (a) Low memory profile, which utilizes shallower neural networks and which is recommended for problems of low complexity or when only low memory resources are available.
  (b) High memory profile, which utilizes deeper and more complex neural networks.
  The framework does not access the memory specifications of users’ computers or cloud servers and hence the memory profile choice is made by the user.
5. Two levels of performance measures are provided in the framework:
  (a) Model-level measures, such as macro and micro F1-score.
  (b) Class level measures, such as recall, precision, and macro average accuracy.
6. Interpretability. The framework displays a random sample of test instances and their corresponding heatmaps for each of the trained neural networks in order to increase the degree to which the user can understand the cause of the network predictions.
7. Warning and comment module. The framework comprises a simple warning and comment module that provides users with guidance and insights about the utilized datasets. Such warnings can help the user to avoid logical and run-time errors that might be hard for users of a non-technical background to solve. Two main types of warnings and comments provided by the framework are:
  (a) The number of classes is checked for both the training and the test sets. A warning is issued if the numbers do not match; otherwise, a comment confirming that the number is equal is rendered.
  (b) A dataset is considered balanced if the SES is higher than a certain threshold (SESTH). The SESTH is obtained empirically. When 0≤SES≤0.5, a warning is issued stating that the dataset is severely imbalanced. On the other hand, a moderate imbalance is issued if 0.5<SES≤0.85.
8. Recommender. Selecting the best performing neural network can be hard for users since there are many class- and model-level metrics. The recommender module recommends the best model based on the highest macro F1 score obtained. The F1 score was chosen because it combines both recall and precision.
9. Neural network training and architecture details. The architecture and number of parameters are displayed for each neural network before training. Moreover, during training, the progress of training and validation accuracy and loss are rendered after each epoch. After training, two figures are depicted: one for the training accuracies against the validation ones, and a second for the training loss against the validation loss values.
10. Advanced options. The user can choose to customize the batch size and the input resolution size or let the framework utilize the default values. The choice of both hyperparameters can affect the training results in image classification applications. If the user chooses to skip tuning those values, then they are set to default values that are commonly used in the literature; for example, the batch size default value is 32.

All the functionalities mentioned above can be set easily by the user through a wizard. Once the user answers all questions in the wizard, the framework runs all functionalities based on the user’s selection.

## 7. Datasets

Three datasets were employed in this work to demonstrate how the proposed framework interacts with two important smart city aspects: smart traffic management and smart healthcare. The dataset can be described in brief in the following list. In addition, more details about the datasets can be found in Section 8.1.

1. Traffic signs dataset. A public dataset of traffic signs that consists of 43 classes of cropped traffic sign images. The dataset consists of 70,989 training instances and 2151 test instances [36].
2. CENPARMI human reticulocyte dataset: This dataset consists of 2461 instances of three main classes: RBCs, reticulocytes, and unknown objects. All smears were inspected under an oil immersion lens of a Nikon ECLIPSE E100 microscope with a 1000× magnification. The microscope was connected to a 14 MegaPixel digital microscope camera that captured the images of the blood smears [37].
3. The feline reticulocytes dataset. The data was collected from two male cats. It consists of whole-smear instances that were taken using a microscope camera and a smartphone camera. The blood cells in this dataset are classified into aggregate reticulocytes, punctate reticulocytes, and erythrocytes [38,39].

## 8. Results and Analysis

To demonstrate the effectiveness and usefulness of the proposed ICF, we utilized five different deep neural networks on three different datasets. This section presents the obtained results at four levels: EDA results, training results, analyzer results, and recommender results.

### 8.1. EDA Results

A key aim of this framework is to help non-technical users in developing a “sense” of their data and ultimately make educated and data-driven decisions. Therefore, EDA is a key component of this framework, as it is designed to help users better understand their data. In this module, a sample of instances, some statistics about the datasets, and the distribution of classes in the datasets are calculated and plotted. In the following subsections, we present some of the module results.

**Figure 4:**
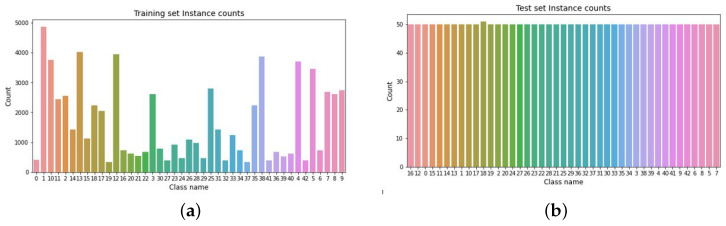
Instances count for the traffic sign dataset. (**a**) Training instance count for the traffic sign dataset. (**b**) Test instance count for the traffic sign dataset.

**Traffic signs dataset.** The distribution of instances in the traffic signs dataset is shown in Figure 4. It shows the instance count for the training and test sets.

Table 2 depicts the Shannon entropy score (SES) results for the traffic signs dataset. It shows that both sets (training and test) are balanced.

**Figure 5:**
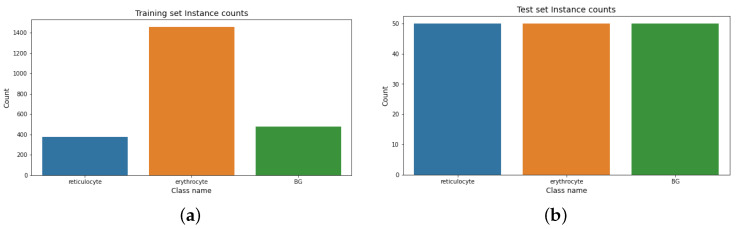
Instances count for the human reticulocyte dataset. (**a**) Training instance count for the human reticulocyte dataset. (**b**) Test instance count for the human reticulocyte dataset.

**Human reticulocyte dataset.** The distribution of instances in the human reticulocyte dataset is shown in Figure 5. It shows the instance count for the training and test sets.

Table 3 depicts the Shannon entropy score (SES) results for the dataset. It shows that the test set is balanced, while the training set is moderately imbalanced.

**Figure 6:**
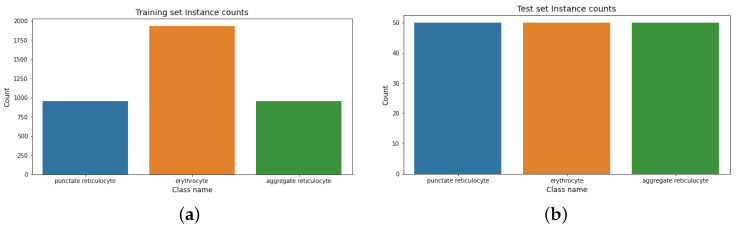
Instances count for the feline reticulocyte dataset. (**a**) Training instance count for the feline reticulocyte dataset. (**b**) Test instance count for the feline reticulocyte dataset.

**Feline reticulocyte dataset.** The distribution of instances in the feline reticulocyte dataset is shown in Figure 6. It shows the instance counts for the training and test sets.

Table 4 depicts the Shannon entropy score (SES) results for the feline reticulocyte dataset. It shows that both sets (training and test) are balanced.

### 8.2. Training Results

**Figure 7:**
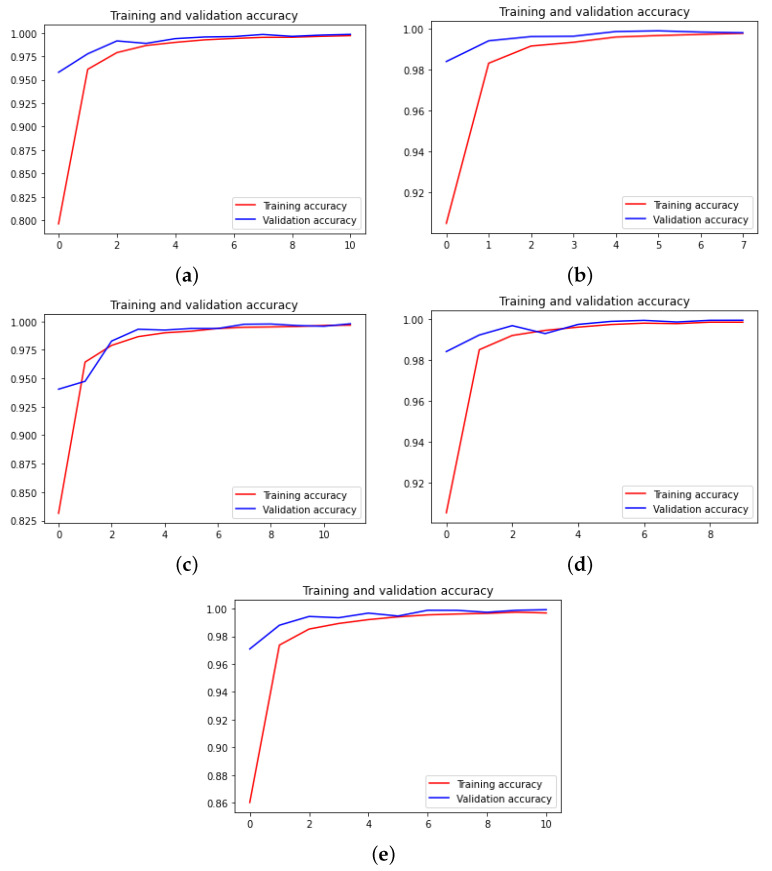
Accuracy curves for the traffic signs dataset. (**a**) Xception. (**b**) DensNet. (**c**) MobileNet. (**d**) ResNet. (**e**) VGG.

**Figure 8:**
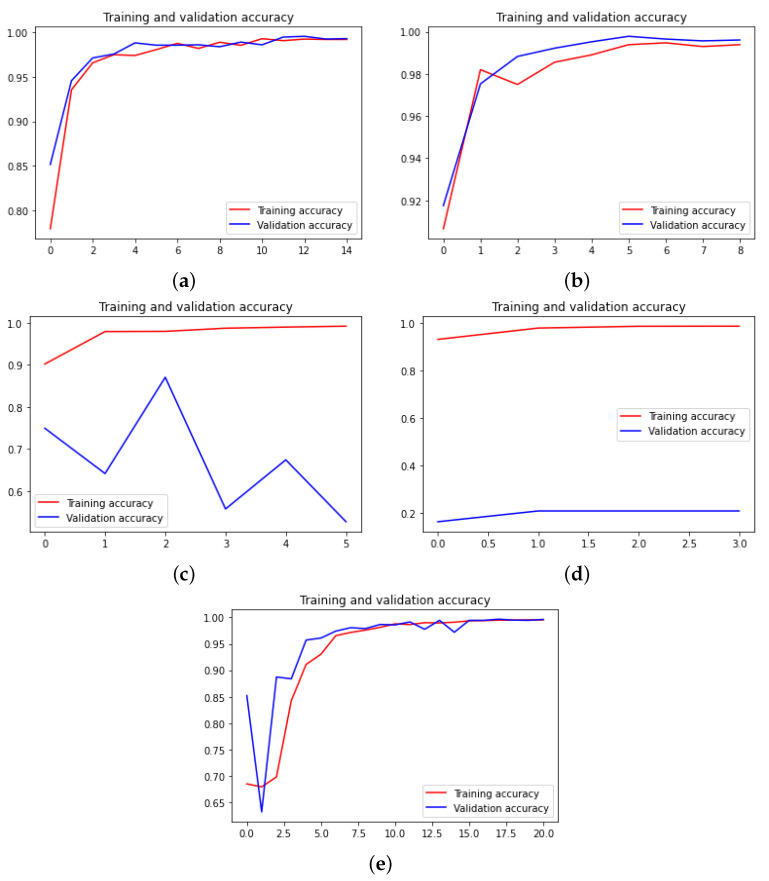
Accuracy curves for the human reticulocyte dataset. (**a**) Xception. (**b**) DensNet. (**c**) MobileNet. (**d**) ResNet. (**e**) VGG.

**Figure 9:**
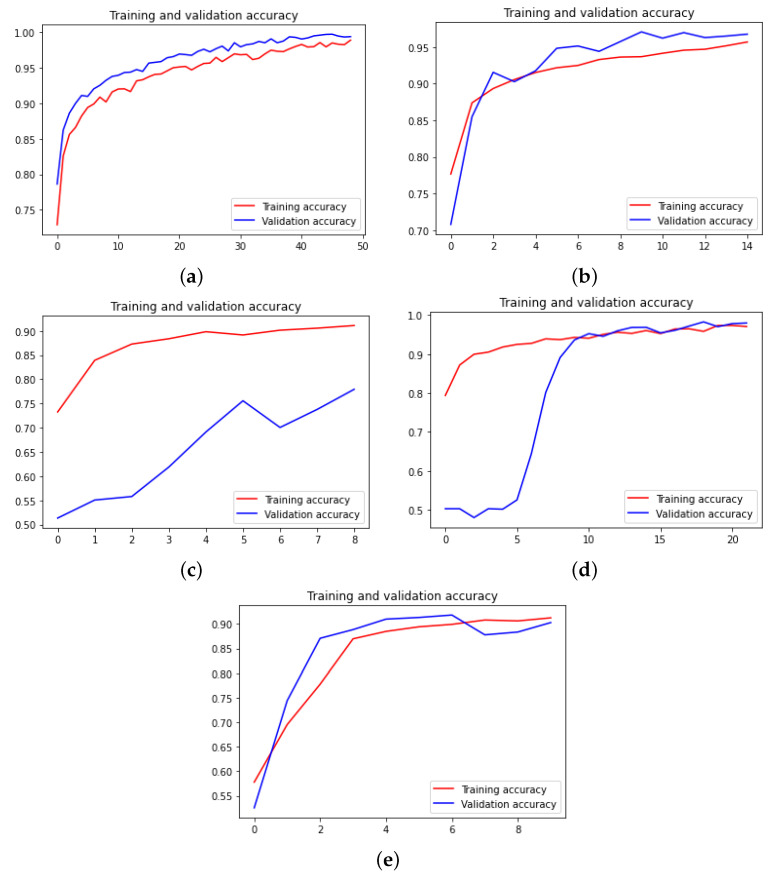
Accuracy curves for the feline reticulocyte dataset. (**a**) Xception. (**b**) DensNet. (**c**) MobileNet. (**d**) ResNet. (**e**) VGG.

Now, we depict the training accuracy curves for all neural networks and for each dataset in Figure 7, Figure 8 and Figure 9. The loss curves are the inverse of the accuracy ones; hence, the loss curves are not depicted.

### 8.3. Analyzer Results

The results in this section are reported at different levels: the model level and the class level. For the class level, we obtained precision, recall, and F1-score. For the model level, we obtained and report accuracy (both macro average and weighted average), macro F1-score, and micro F1-score. Due to the large number of classes in each dataset, the class level results are omitted and only a general description of the results is provided.

Table 5 summarizes the model-level results obtained for the traffic signs dataset. Table 6 summarizes the results obtained for the human reticulocyte dataset. Furthermore, Table 7 summarizes the results obtained for the feline reticulocyte dataset.

**Figure 10:**
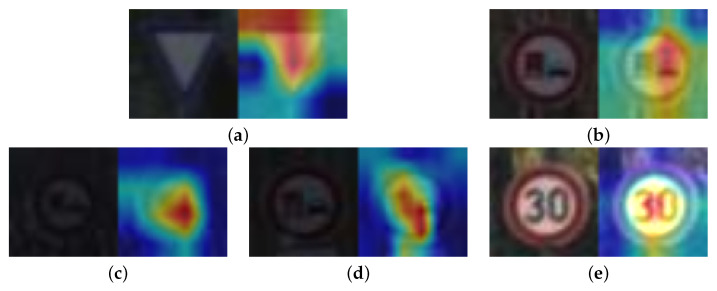
Sample heatmaps for the traffic signs dataset. (**a**) Densenet HeatMap. (**b**) MobileNet HeatMap. (**c**) ResNet HeatMap. (**d**) VGG HeatMap. (**e**) Xception HeatMap.

**Figure 11:**
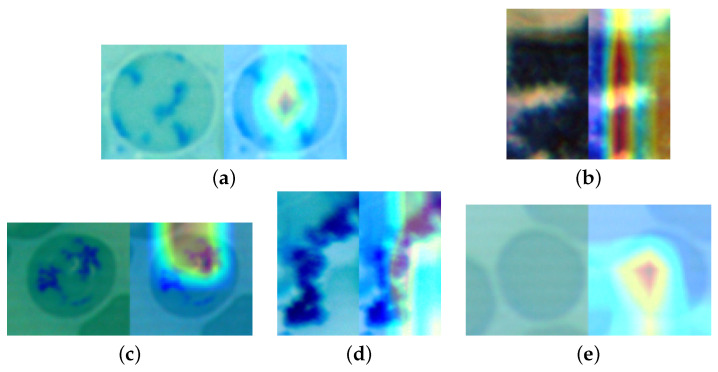
Sample heatmaps for the human reticulocyte dataset. (**a**) Densenet HeatMap. (**b**) MobileNet HeatMap. (**c**) ResNet HeatMap. (**d**) VGG HeatMap. (**e**) Xception HeatMap.

**Figure 12:**
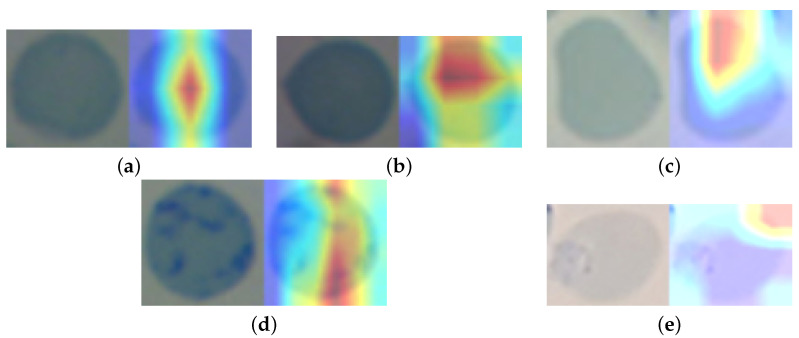
Sample heatmaps for the feline reticulocyte dataset. (**a**) Densenet HeatMap. (**b**) MobileNet HeatMap. (**c**) ResNet HeatMap. (**d**) VGG HeatMap. (**e**) Xception HeatMap.

Moreover, we show a sample of the heatmaps used by the models to identify which features were more helpful in the classification process. The samples are shown in Figure 10, Figure 11 and Figure 12.

### 8.4. Recommender Results

Finally, the recommender component evaluates all results and lists suggestions and recommendations to the end-user. It is worth mentioning that it is common to have varying results from different types of neural networks, as each neural network uses a different type of layers and convolutions and, hence, varying results are obtained using the proposed framework [40]. For the traffic signs dataset, the VGG16 was recommended, whereas for the human reticulocyte dataset, the ResNet model was recommended. Finally, for the feline reticulocyte dataset, the DensNet121 model was recommended.

## 9. Conclusions and Future Work

To alleviate the increasing challenges that face urban spaces at the present time and in the future, one must invest in smart cities. The concept of a smart city has yet to be well defined; however, it encompasses any attempt to utilize and employ technological solutions to face existing problems. Hence, the stakeholders involved in smart cities are many and they come from different backgrounds. In this work, we first tried to shed light on the definition of a smart city and what it means from different perspectives. Then, we proposed a framework and model to design a smart city from scratch that achieves its goals and opens the door to better communication and understanding between all stakeholders. The main design concept is to match the high-level goals/objectives with a design that can fit all these demands. For this, the city is divided into zones for better management and to reduce overhead (such as energy and transportation).

The main limitation of this work is that the image classification framework does not have access to users’ hardware specifications; hence, the memory profile choice has to be made by the framework user.

We note that no single system can achieve all these goals, and a smart city must utilize many smart systems to do so. The smart systems must take into consideration the existing infrastructure (i.e., people/employees, available hardware, regulations). One key challenge here is the lack of specialized personnel to deal with new and emerging technologies and systems such as deep learning and data sciences. To prove the design concept proposed in this work, we designed and implemented a smart image classification framework (ICF) that can be used by non-technical personnel and that employs many deep network models to classify images. The ICF provides many features such as transfer learning, results transparency, exploratory data analysis, and memory profiling. To demonstrate the effectiveness of the proposed system, we tested the ICF on three different datasets spanning different possible applications in smart cities, in particular, traffic and health applications. Currently, ICF supports five different deep networks. The obtained results show that the system behaves as expected and can be fully utilized by non-technical users. For future work, we propose to extend the proposed ICF system to handle video data formats and support other deep networks.

## Figures and Tables

**Table 1:** Variations in smart/digital city definitions.

Theme	Def	Background	Focus	Year	Ref
Digital city	Web-based urban information systems and virtual communities	Urban planning	ICT and society	2005	[8]
Digital city	Online commerce for retail shops	Retail	Web	2018	[9]
Digital city	Exploration of cyberspace	Social and ICT	Web	2017	[10]
Smart city	Exploitation of physical space	Social and ICT	Sensors	2017	[10]
Smart city	The application of massive amounts of digital data collected about society as a means to rationalize the planning and management of cities	Geographical	Data	2014	[11]
Smart city	The ability to promote economic growth	B	T		[11]
Smart city	A technocratic view of urban management and government	Public policy	Social	2016	[12]
Smart city	A city that makes optimal use of all interconnected information available today to better understand and control its operations and optimize the use of limited resources	Commercial	Technology	2011	[13]

**Table 2:** SES results for the traffic sign dataset.

Set	SES	Remarks
**Training**	0.9217880704013425	The training set is balanced
**Test**	0.99999880085778	The test set is balanced

**Table 3:** SES results for the human reticulocyte dataset.

Set	SES	Remarks
**Training**	0.830234057739414	MODERATE IMBALANCE ALERT
**Test**	0.9999999999999998	The test set is balanced

**Table 4:** SES results for the feline reticulocyte dataset.

Set	SES	Remarks
**Training**	0.9444029604077115	The training set is balanced
**Test**	0.9999999999999998	The test set is balanced

**Table 5:** Summary of results for traffic signs dataset.

Model	Accuracy	Macro F1-Score	Micro F1-Score	Macro Avg Precision	Macro Avg Recall	Macro Avg F1 Score
**Xception**	1.0	0.998	0.998	1.0	1.0	1.0
**Densenet**	1.0	0.9976	0.9976	1.0	1.0	1.0
**MobileNet**	1.0	0.9976	0.9976	1.0	1.0	1.0
**VGG**	1.0	0.999	0.9999	1.0	1.0	1.0
**ResNet**	1.0	0.998	0.998	1.0	1.0	1.0

**Table 6:** Summary of results for human reticulocyte dataset.

Model	Accuracy	Macro F1-Score	Micro F1-Score	Macro Avg Precision	Macro Avg Recall	Macro Avg F1 Score
**Xception**	0.97	0.9733	0.9733	0.97	0.97	0.97
**Densenet**	0.99	0.99	0.9933	0.9933	0.99	0.99
**MobileNet**	0.74	0.7031	0.74	0.81	0.74	0.7
**VGG**	0.99	0.9866	0.9866	0.99	0.99	0.99
**ResNet**	0.33	0.1666	0.3333	0.11	0.33	0.17

**Table 7:** Summary of results for feline reticulocyte dataset.

Model	Accuracy	Macro F1-Score	Micro F1-Score	Macro Avg Precision	Macro Avg Recall	Macro Avg F1 Score
**Xception**	0.91	0.9061	0.9066	0.91	0.91	0.91
**Densenet**	0.93	0.9335	0.9333	0.94	0.93	0.93
**MobileNet**	0.71	0.7043	0.7067	0.76	0.71	0.7
**VGG**	0.92	0.92	0.92	0.92	0.92	0.92
**ResNet**	0.89	0.891	0.893	0.9	0.89	0.89
